# Mapping DEHP to the adverse outcome pathway network for human female reproductive toxicity

**DOI:** 10.1007/s00204-022-03333-y

**Published:** 2022-07-05

**Authors:** Kristina Pogrmic-Majkic, Dragana Samardzija Nenadov, Biljana Tesic, Svetlana Fa Nedeljkovic, Dunja Kokai, Bojana Stanic, Nebojsa Andric

**Affiliations:** Department of Biology and Ecology, Faculty of Sciences, University of Novi Sad, Trg Dositeja Obradovica 2, 21000 Novi Sad, Serbia

**Keywords:** Adverse outcome pathway (AOP), AOP network, Female reproductive toxicity, DEHP

## Abstract

Adverse outcome pathways (AOPs) and AOP networks are tools for mechanistic presentation of toxicological effects across different levels of biological organization. These tools are used to better understand how chemicals impact human health. In this study, a four-step workflow was used to derive the AOP network of human female reproductive toxicity (HFRT-AOP) from five AOPs available in the AOP-Wiki and ten AOPs obtained from the literature. Standard network analysis identified key events (KEs) that are point of convergence and divergence, upstream and downstream KEs, and bottlenecks across the network. To map di-(2-ethylhexyl) phthalate (DEHP) to the HFRT-AOP network, we extracted DEHP target genes and proteins from the Comparative Toxicogenomic and the CompTox Chemicals Dashboard databases. Enriched GO terms analysis was used to identify relevant biological processes in the ovary that are DEHP targets, whereas screening of scientific literature was performed manually and automatically using AOP-helpFinder. We combined this information to map DEHP to HFRT-AOP network to provide insight on the KEs and system-level perturbations caused by this endocrine disruptor and the emergent paths. This approach can enable better understanding of the toxic mechanism of DEHP-induced human female reproductive toxicity and reveal potential novel DEHP female reproductive targets for experimental studies.

**Supplementary Information:**

The online version contains supplementary material available at 10.1007/s00204-022-03333-y.

## Introduction

An adverse outcome pathway (AOP) represents a sequential series of biological effects linking molecular initiating event (MIE) and an adverse outcome (AO) relevant to risk assessment. MIE and AO are linked with a series of intermediate steps known as the key events (KEs) (Vinken 2013). In other words, an AOP is a simplified depiction of complex toxicological processes in a linear and modular format starting with an MIE and ending with an AO (Halappanavar et al. 2020). The AOP concept can be used for broad purposes including the establishment of structure–activity relationships, the development of novel in vitro toxicity screening tests, and the elaboration of prioritization strategies (Vinken 2013). Moreover, it can be useful in identifying data gaps and uncertainties in the pathway linking exposure to the molecular and cellular response and then to the potential AO (Leist et al. 2017).

Pathways are often interconnected and perturbations in one pathway will lead to perturbations of the connected branches, thus forming a more complex and comprehensive network—an AOP network. An AOP network is, therefore, defined as an assembly of two or more AOPs that share one or more KEs, including specialized KEs such as MIEs and AOs (Knapen et al. 2018). The AOP network more faithfully depicts the path to the disease processes; hence, it is more applicable to real-life scenarios (Halappanavar et al. 2020). Based on the results of the Horizon Scanning exercise sponsored by the Society of Environmental Toxicology and Chemistry that focused on advancing the AOP framework, the development of guidance related to AOP network development was identified as a critical need (Knapen et al. 2018). In support of the AOP network development, the critical mass of individual AOPs from the AOP-Wiki has been compiled, thus allowing the construction, analysis, and testing of the AOP networks (Villeneuve et al. 2018). Two AOP network case studies, namely the AOP network for metabolic disorders mediated by hepatic steatosis and the AOP network related to disruption of the thyroid axis (Knapen et al. 2018), followed by the CYP19-AOP and T4-AOP networks (Villeneuve et al. 2018) nicely illustrate the concept, development, and application of the AOP networks. These studies further catalyzed the development of AOP networks for human neurotoxicity (Spinu et al. 2019) and hepatotoxicity (Arnesdotter et al. 2021), and the AOP network relevant to nanomaterials (Halappanavar et al. 2020). All these derived AOP networks further solidify the potential of AOP networks to address the knowledge gaps, prioritize the testing strategies, and support hazard characterization and chemical risk assessment.

Following a possible link between exposure to environmental chemicals and alterations in human and wildlife reproduction and development, endocrine disruptors (EDs) became a public and regulatory concern (Browne et al. 2020). The Organisation for Economic Co-operation and Development (OECD) started to investigate test methods that could be used to detect and characterize hazards posed by EDs; however, identification of an ED requires evaluation of the data obtained from multiple test methods, integration of information regarding a chemical’s interaction with a target (e.g., cellular receptor or key enzyme) and the manifestation of that interaction at higher levels of biological complexity (e.g., organ or organism) (Browne et al. 2020). To facilitate identification of EDs and their toxicological and mechanistic effects, AOP networks related to endocrine disruption started to emerge (Knapen et al. 2018; Ravichandran et al. 2022; Villeneuve et al. 2018). These endocrine disruption-related AOP networks help in identification of events, critical paths, and unexpected links between individual AOPs capturing varied endocrine adverse effects; however, these AOP networks cover a broad spectrum of endocrine disruptive events and do not capture specific mechanisms related to female reproductive toxicology.

Di-(2-ethylhexyl) phthalate (DEHP; CAS# 117-81-7) is a powerful ED that belongs to the group of phthalates. The European Commission’s Strategy for Endocrine Disruptors has developed the Priority list of chemicals for further evaluation of their endocrine disrupting effects (https://ec.europa.eu/environment/chemicals/endocrine/strategy/substances_en.htm). According to Annex 1 (Candidate list of 553 substances) of the Priority list, DEHP is assigned to Category 1—Evidence of endocrine disrupting activity in at least one species using intact animals (https://ec.europa.eu/environment/archives/docum/pdf/bkh_annex_01.pdf). Moreover, in Annex 15—List of 66 substances with classification high, medium, or low exposure concern, DEHP is categorized as a chemical of high concern (https://ec.europa.eu/environment/archives/docum/pdf/bkh_annex_15.pdf). Human exposure to DEHP arises mainly from ingestion, inhalation, and dermal absorption; however, food packaged in plastic/PVC materials can also contribute to the exposure (Wang et al. 2019). DEHP was found in serum and urine of children, young girls, and women (Colon et al. 2000; Hogberg et al. 2008; Kardas et al. 2016; Sun et al. 2016), in endometrial tissues and breast milk (Colon et al. 2000; Sun et al. 2016), whereas DEHP metabolites were found in follicular fluid in the nM range (Krotz et al. 2012). One of the main targets of DEHP is the female reproductive system. Human epidemiological studies show an association between DEHP exposure and adverse reproductive outcomes in women, such as an increased risk of miscarriage, a decreased pregnancy rate in female factory workers (Heudorf et al. 2007), and endometriosis (Kim et al. 2015). Moreover, an association between the levels of urinary phthalate metabolites in couples involved in the in vitro fertilization (IVF) procedure shows that phthalate exposure adversely affects the IVF outcome, increases the risk of biochemical pregnancy, failed clinical pregnancy, and decreases the percentage of live births (Al-Saleh et al. 2019). Animal studies further confirmed negative effects of DEHP on the female reproductive system by showing that this ED can prevent proliferation of granulosa cells and promote apoptosis (Liu et al. 2018), inhibit steroidogenesis (Chiang et al. 2020) and development of antral follicles in the offspring (Liu et al. 2021a), extend estrous cycle duration (Adam et al. 2021), and suppress ovulation (Davis et al. 1994). In vitro exposure of granulosa cells to DEHP caused inhibition of steroid production (Ernst et al. 2014; Guerra et al. 2016) and promotion of apoptosis (Jin et al. 2019; Tripathi et al. 2019a). Several studies have demonstrated the potential mechanism for DEHP-mediated alterations in ovarian function including oxidative stress (Tripathi et al. 2019a), expression of the peroxisome proliferator-activated receptor (PPAR) alpha and gamma (Ernst et al. 2014), and estrogen receptor (ER) (Mu et al. 2015). Besides a number of diverse female reproductive targets of DEHP, concentrations/doses of this ED capable of eliciting the effect on female reproductive system are also diverse. These concentrations range from high (Tripathi et al. 2019a), to the concentrations that are in line with oral tolerable intake for DEHP (Lu et al. 2019), or the concentrations reported in the blood of patients that were long-term exposed to DEHP-containing medical equipment (Liu et al. 2017) and the concentrations that are near those detected in human blood, i.e., from 1 to 100 nM (Guerra et al. 2016; Jin et al. 2019). It is obvious that the available data on the effects of DEHP on female reproductive system are diverse and scattered, thus preventing a systematic organization of the KEs across different levels of biological organization involved in the adverse effects of DEHP in female reproductive system.

The aims of this study were: (i) to derive the AOP network for human female reproductive toxicity based on the available AOPs in AOP-Wiki or the available literature and characterize the network using analytics proposed by (Knapen et al. 2018; Villeneuve et al. 2018), and (ii) to map DEHP to the AOP network for human female reproductive toxicity by taking into consideration dose- and time-dependent effects. This comprehensive analysis will provide a better understanding of the mechanism of DEHP action across different levels of biological organization, thus identifying potential gaps and prioritizing experimental research.

## Materials and methods

### Dataset

The dataset for the construction of the HFRT-AOP network was obtained from two sources. The first source, AOP-Wiki (https://aopwiki.org), was manually examined to select the linear AOPs related to human female reproductive toxicity. The following information for each AOP was collected: AOP ID, KE type (i.e., MIE, KE, AO), KEs (KEIn and KEOut), adjacency of the relationship between the pair of KEs, the qualitative weight of evidence (WoE) (i.e., low, moderate, high), and progress through the OECD review (Arnesdotter et al. 2021; Spinu et al. 2019). The data were collected on March 09, 2022. Four out of five individual AOPs obtained from the AOP-Wiki have “under development” status and should therefore be considered as putative. The second dataset was obtained from the paper of Johansson et al. (2020). Their study contains 10 putative adverse outcome networks (pAOP) related to human female reproduction organized into four AOP networks and one disconnected AOP. The title, molecular, cellular/tissue, organ, and organism events for each individual pAOP were collected.

### AOP network construction

The AOP network for human female reproductive toxicity was constructed following the general design principle described elsewhere (Spinu et al. 2019).

#### Step 1

The purpose of this study was to identify the most common and highly connected KEs in the HFRT-AOP network, which was used to analyze the critical KEs in the network.

#### Step 2

To construct the HFRT-AOP network, we examined several elements in AOPs related to female reproduction available at the AOP-Wiki: sex, taxonomy, life stage applicability of the individual linear AOPs, and the KE titles related to human female reproductive toxicity. We only selected AOPs that are applicable for sex—female, species—human or rodent, and stage—adult. Although related to female reproductive toxicology and included in another AOP network (Villeneuve et al. 2018), we have excluded several AOPs for the following reasons: taxonomy applicability—fish species and KE 285—decreased vitellogenin synthesis in the liver (AOP ID 25*,* AOP ID 23, AOP ID 122, AOP ID 123, AOP ID 30), taxonomy applicability—fish species and KE 307—increased vitellogenin synthesis in the liver (AOP ID 29), taxonomy applicability—fish species and KE 673—reduced spawning behavior (AOP ID 100), taxonomy applicability—fish species and KE 1004—posterior swim bladder inflation (AOP ID 155) and KE 1007—reduced, anterior swim bladder inflation (AOP ID 156), and taxonomy applicability—*Caenorhabditis elegans* with unspecific sex *(*AOP ID 207). Although related to human female reproduction, AOP ID 165 was excluded from the network construction since it contains only non-adjacent connections between some of the KEs and between the KEs and the AOs. We also included the pAOPs published in the paper of Johansson et al. (2020). A manual analysis of this study showed that all pAOPs were related to human female reproduction. All the information about individual AOPs used to derive the HFRT-AOP network are presented in Table 1 and Supplementary Material 1, Table S1. Titles of biological events in the pAOPs were grouped into “Molecular”, “Cellular/Tissue”, “Organ”, and “Organism” categories. For purpose of harmonization, these biological events were re-grouped as follows: MIE (“Molecular”), KE (“Cellular/Tissue”), and AO (“Organ”) (Supplementary Material 1, Table S2). pAOP7 represents an adaptation of AOP7 from the AOP-Wiki and was already included in the network construction in the first dataset.

**Table 1:** Fifteen AOPs related to human reproductive toxicity

AOP-Wiki ID	AOP title	Sex/taxonomy/life stage applicability	OECD status (as of February 2022)
7	Aromatase (Cyp19a1) reduction leading to impaired fertility in adult female	Adult/*Homo* *sapiens*, *Rattus* *norvegicus*, *Mus* *musculus*/female	EAGMST under review
345	Androgen receptor (AR) antagonism leading to decreased fertility in females	–/–/–	–
153	Aromatase inhibition leading to ovulation inhibition and decreased fertility in female rats	–/–/–	–
398	Inhibition of ALDH1A (RALDH) causing reduced all-trans retinoic acid levels leading to impaired fertility in females	During development and at adulthood/*Homo* *sapiens*, *Rattus* *norvegicus*, *Mus* *musculus*/female	–
238	Deposition of energy leading to population decline via ovarian follicle breakdown	Adult/*Mus* sp., *Daphina* *magna*/female	–

pAOP (Johansson et al. 2020)	AOP title
1	Reduced retinoic acid levels lead to reduced follicle pool and impaired fertility in females
2	Impaired GPER activity leads to reduced follicle pool and impaired fertility in females
3	Disrupted meiotic division leads to impaired oocyte quality and impaired fertility in females
4	Disrupted ESR and AHR signaling leads to disturbed primordial follicle formation and impaired fertility in females
5	Disrupted AHR signaling leads to follicle atresia and premature ovarian insufficiency
6	Early PI3K activation leads to premature ovarian insufficiency
8	Decreased INSL3 synthesis leads to reduced follicle maturation and impaired fertility in females
9	Disrupted AR activity leads to altered follicle growth and impaired fertility
10	DNA methylation in LHCGR promoter leads to disturbed ovulation and impaired fertility in females

#### Step 3

The AOPs identified according to the criteria in Step 2 were evaluated and collected manually in an Excel spreadsheet. In some cases, KEs from different AOPs referring to the same biological processes were given different titles. These KEs were analyzed, grouped, and renamed under a common KE title. All amendments to the KE titles are described in the Excel spreadsheet (Supplementary Material 1, Table S3).

#### Step 4

Cytoscape 3.8.0 (https://cytoscape.org/), an open-source software platform, was used to model the AOP network. The HFRT-AOP network was generated as a directed graph where each unique KE is represented by a single node, and the KE relationships (KERs) are represented by edges (Supplementary Material 1, Table S4). NetworkAnalyzer 4 App, pre-installed in the Cytoscape software, was used to analyze the derived network. The nodes (MIEs, KEs, and AOs) were manually positioned to maximize readability. Information regarding the WoE, adjacency, and the type of KE was added to further define the visual attributes of the AOP network. The duplicate edges and self-loops were removed and the KERs shared by more than one AOP were shown by a single arrow.

### Network analysis

The level of degree, betweenness centrality, and eccentricity were used to analytically characterize the network due to their ability to quantify the position of a single KE in relation to its neighboring KEs in the network in Cytoscape NetworkAnalyzer 4.4.6 App. Indegree and outdegree scores as a measure of KEs convergence or divergence were obtained by analyzing the network as a direct graph. Betweenness centrality score shows nodes that lie on a high proportion of paths between other nodes in the network. The KEs with a high betweenness centrality score may represent critical control KEs within the network, whereas the eccentricity score can identify the most upstream and downstream KEs in the network.

### Linking DEHP with biological effects

To identify genes and proteins known to be associated with DEHP exposure, we compiled information from two publicly available databases. First, we extracted information from the Comparative Toxicogenomic Database (CTD), which provides manually curated information about chemical–gene/protein interactions, chemical–disease, and gene–disease relationships (http://ctdbase.org/). The literature search in CTD was conducted in May 2021. The full chemical name “Diethylhexyl Phthalate” was an input in the “Chemicals” search box and the references were retrieved and downloaded for further evaluation. In this study, only experimental research papers on DEHP-induced toxicity were selected. The exclusion criteria were taken from elsewhere (Huang et al. 2021), with some modifications. These criteria were (1) toxicity after co-exposure with other chemicals; (2) epidemiological studies in which human blood or urine samples were used; (3) without available full text or written in language other than English; and (4) monitoring of DEHP concentration in the environment. The filtered references were further marked and selected based on the ovaries as a target organ. If in vitro cell culture was used to analyze the effects of DEHP, the cell origin was taken as the target organ of DEHP exposure. To collect more information about association of DEHP with proteins, the CompTox Chemicals Dashboard database was used (https://CompTox.epa.gov/dashboard/, accessed on May 2021). It is a publicly available web-based application developed by the US Environmental Protection Agency to provide access to chemistry, toxicity, and exposure information for  ~ 900,000 chemicals (Williams et al. 2021).

### Bioinformatics analysis

All genes retrieved from the literature search were manually inspected, deduplicated, and pooled. The genes were used as an input for bioinformatics analysis in the Database for Annotation, Visualization and Integrated Discovery (DAVID v6.8) and the enriched Gene Ontology (GO) term “Biological Processes” (BP) were extracted. The data were presented using the “ggplot2” package in the R software.

### Literature search

The literature search was conducted in two ways with the aim to capture as much information as possible to link DEHP to the HFRT-AOP network. First, an automatic search was performed using the recently developed AOP-helpFinder tool (Jornod et al. 2021). This tool is a hybrid approach that combines text mining procedures and graph theory to identify the linkage between a stressor and biological events. For search, we used several synonyms for DEHP and biological targets identified in the CTD and the CompTox database (Supplementary Material 1, Table S5). The second search was conducted using PubMed database to screen information on DEHP and human or rodent female reproductive system. The screen was made using the keywords “DEHP” and “ovary”. The search results were analyzed by the keywords in the title and article abstract. If links were mentioned in the summary, the article was read to find the relevant information and retrieve it.

## Results and discussion

### Development of the HFRT-AOP network

To better understand the sequential series of KEs involved in DEHP-induced female reproductive system toxicity, we first generated the HFRT-AOP network from individual AOPs related to human female reproduction. The AOP networks related to female reproduction such as the CYP19-AOP network (Villeneuve et al. 2018) and the AOP network based on five reproductive and developmental toxicity-related AOPs (Knapen et al. 2015) were previously generated; however, the HFRT-AOP network derived in this study was somewhat different. We used only AOPs that were relevant to human female reproductive toxicity. We identified five AOPs in the AOP-Wiki that fulfill the criteria described in the Material and Methods section. Since the amount of information in the AOP-Wiki related to human female reproduction was limited, we have decided to populate individual AOPs from the AOP-Wiki with 10 published pAOPs relevant to human female reproduction (Johansson et al. 2020). The information about selected AOPs used for the construction of the HFRT-AOP network is presented in Table 1 and Supplementary Material 1, Table S1.

We found that different AOPs contain KEs that indicate the same process but were labeled differently. For example, MIE 408 in AOP ID 7 “Reduction in ovarian granulosa cells aromatase (Cyp19a1)” and MIE 964 in AOP ID 153 “Inhibition of aromatase enzyme chemical exposure during critical window of estrous cycle b” refer to the same process, inhibition of aromatase, but are titled differently. The decrease in estradiol (E2) production by granulosa cells is referred by KE 3 in AOP ID 7 and KE 965 in AOP ID 153, whereas impaired fertility has been addressed by AO 328 in AOP ID 238 “Decrease, fecundity” and AO 1277 in AOP ID 207 “Reproductive failure”. There were also similar inconsistencies in MIEs’, KEs’, and AOs’ annotation between the AOPs from the AOP-Wiki and the pAOPs obtained from the paper of Johansson et al. 2020. Since these inconsistencies in annotation can have a major impact on network construction, topology, and analytics (Arnesdotter et al. 2021), we have reviewed, grouped, and renamed them under common titles (Supplementary Material 1, Table S3).

**Fig. 1:**
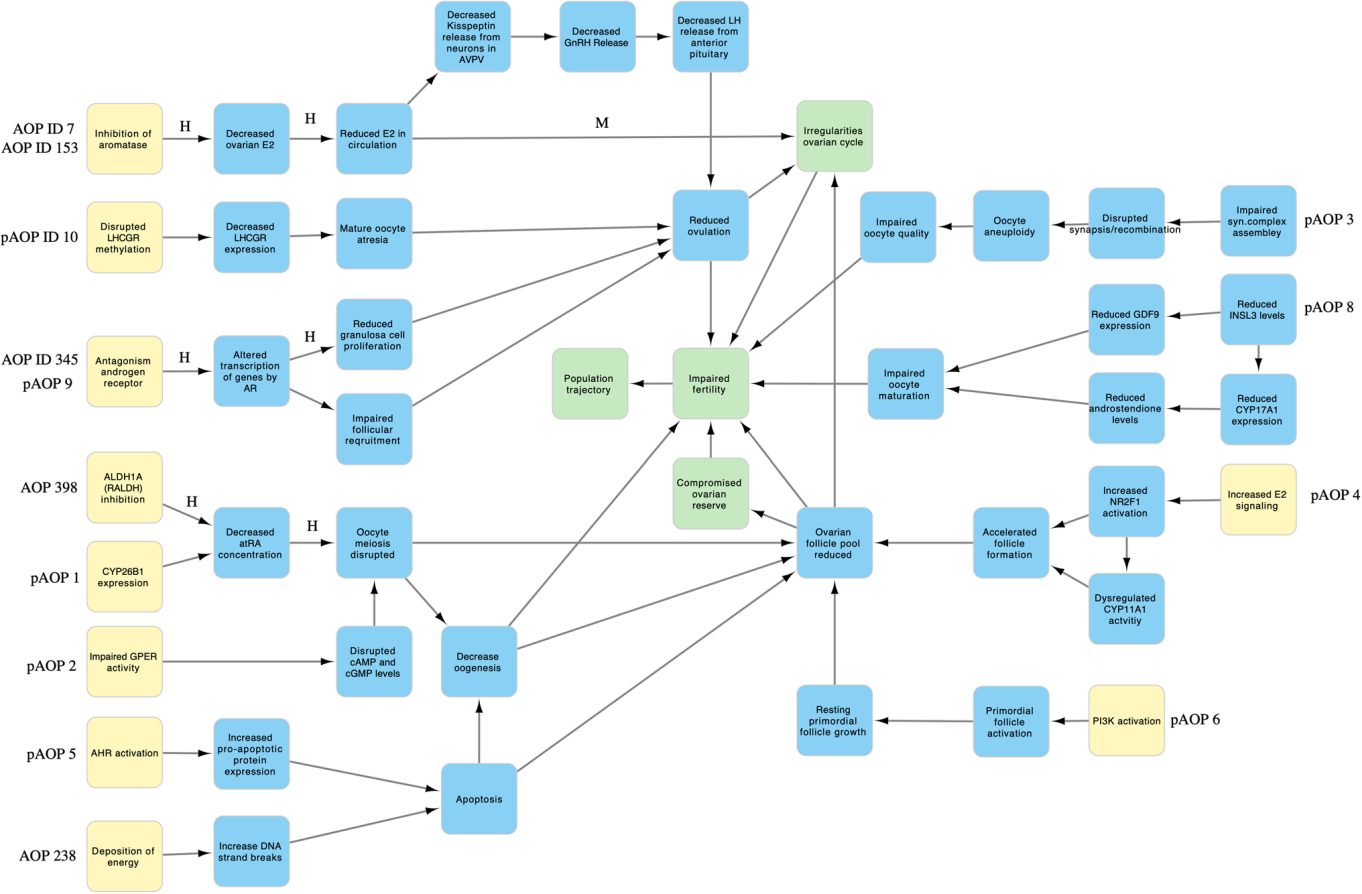
The network of 15 AOPs for human female reproductive toxicity. Yellow squares indicate MIEs, blue squares indicate KEs, and green squares indicate AOs. KER label indicates the strength of evidence as defined by the AOP author in the AOP-Wiki, where *H* high, *M* medium. No label indicates lack of information in the AOP-Wiki. A dashed line indicates a non-adjacent relationship

The derived HFRT-AOP network is shown in Fig. 1. To better understand the system-level effects, we have categorized the HFRT-AOP network into four system-level subnetworks based on the shared KEs. The subnetwork “Reduced ovulation-dependent fertility impairment” includes AOPs ID 7, 153, and 345, and pAOPs 9 and 10. These AOPs converge to the KE “Reduced ovulation”, which leads to the AO “Impaired fertility” directly or through the AO “Irregularities, ovarian cycle”. It can be also noticed that different biological events can lead to the KE “Reduced ovulation”, such as reduction in E2 production, alterations in androgen receptor (AR) function, or decrease in luteinizing hormone/choriogonadotropin receptor (LHCGR) methylation. The AOPs ID 398 and 238, and pAOP 1, 2, 4, 5, and 6 share the KE “Ovarian follicle pool reduced” and therefore can be grouped in the subnetwork “Folliculogenesis-related fertility impairment”. This subnetwork shares only the AO “Irregularities, ovarian cycle” with the subnetwork “Reduced ovulation-dependent fertility impairment”. The two smaller subnetworks are related to “Oocyte-dependent fertility impairment” and encompass pAOP 8 that leads to the KE “Impaired oocyte maturation” and pAOP 3 that leads to the KE “Impaired oocyte quality”. Although these two subnetworks are related to the oocyte dysfunction, they do not share any MIEs and KEs. Categorization of an AOP network into subnetworks has been previously done for the AOP network for endocrine-mediated perturbations (Ravichandran et al. 2022). Determination of subnetworks in this study was based on the shared KEs between the AOPs; however, harmonization of KEs, which is based on the researcher’s presumption of the same/similar biological process between different AOPs, may affect determination of subnetworks. For example, the AO “Decreased fertility, Reduced number of oocytes ovulated” was renamed to “Reduced ovulation” and annotated as the KE to harmonize with the KE “Reduced ovulation of oocytes” or “Reduced ovulation” in pAOP 9 and 10. In the study of Johansson et al. 2020, pAOPs 1, 2, and 3 were placed in the common network “Disrupted meiosis leading to impaired fertility”, whereas in this study, pAOPs 1 and 2 converge to the same KE “Ovarian follicle pool reduced” and do not share any KEs with pAOP3. It can be argued that the KEs “Impaired oocyte quality”, “Impaired oocyte maturation”, and “Oocyte meiosis disrupted” refer to the same process and should be placed under a common subnetwork; however, the construction of the network consisting of 15 individual AOPs and harmonization of nodes may lead to different AOP segregation and, therefore, subnetwork annotation.

### The HFRT-AOP network analytics

**Fig. 2:**
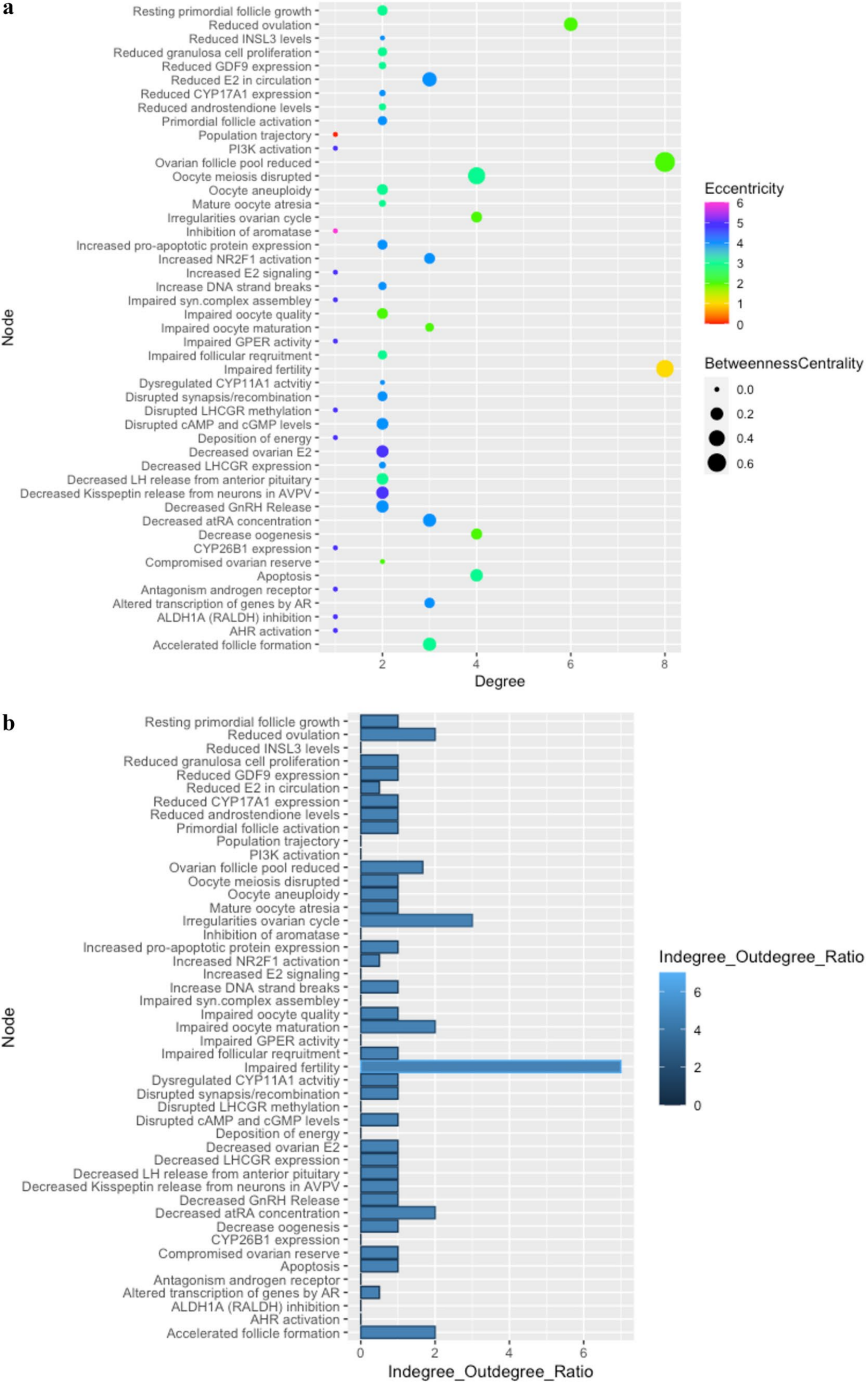
The derived HFRT-AOP network analytics. **A** The nodes degree, eccentricity, and betweenness centrality. **B** The nodes indegree/outdegree ratio

Analysis conducted on the derived HFRT-AOP network is shown in Fig. 2 and Supplementary Material 1, Table S6. The AOs “Impaired fertility” and “Ovarian follicle pool reduced” have the highest level of degree (score 8), followed by the KE “Reduced ovulation” (score 6). Twelve nodes that mostly belong to MIEs showed the degree level of 1. This level of degree is important in the identification of points of convergence and divergence. The point of convergence represents an integrative point that can be affected by several upstream elements. In contrast, a point of divergence is a point of branching off from a common KE to a wider range of possible outcomes (Villeneuve et al. 2018). In a directed AOP network, the node degree can be split into indegree and outdegree, and their ratio places the KE as a point of convergence or divergence. In the HFRT-AOP network, a large number of KEs (51%) show the same number of upstream and downstream connections (Indegree/Outdegree ratio of 1) and 25% of KEs show only outdegree connections. The AO “Impaired fertility” can be considered as a point of convergence since it has the highest Indegree/Outdegree ratio of 7, followed by the AO “Irregularities of ovarian cycle” (Indegree/Outdegree ratio of 3). These AOs represent an apical node and it is expected that more connections converge to them.

The KEs with the lowest Indegree/Outdegree ratio are “Reduced E2 in circulation”, “Altered transcription of genes by AR” and “Increased NR2F1 activation” (ratio of 0.5). These KEs represent a point of divergence and alteration in their function can lead to a change in more than one biological process. Alteration of the KE “Reduced E2 in circulation” can exert effect on hypothalamus causing decrease in the kisspeptin and GnRH levels during ovarian cycle, thus affecting the LH surge necessary to terminate the follicular program and induce ovulation (AOP ID 153). On the other hand, reduced E2 in circulation can impair fertility through direct action on the ovary, thus causing irregularities of the ovarian cycle (AOP ID 7). Similarly, changes in the AR-dependent gene expression are connected with the KE “Reduced ovulation” and the AO “Impaired fertility” through two different KEs: “Reduced granulosa cell proliferation” (AOP ID 345) and “Impaired follicular requirement” (pAOP 9), whereas the KE “Increased NR2F1 activation” is connected to the KEs “Accelerated follicle formation” directly or through the KE “Dysregulated CYP11A1” (pAOP 4).

Further analytics of the AOP network shows that the KE “Ovarian follicle pool reduced” has the highest betweenness centrality (score 0.75), followed by the AO “Impaired fertility” (0.52) and the two KEs “Oocyte meiosis disrupted” (0.50) and “Reduced E2 in circulation” (0.29). These scores suggest that the abovementioned nodes represent the key connector or the bottleneck in the AOP network, the nodes that allow for communication between two neighboring nodes (Pavlopoulos et al. 2011). The KE “Ovarian follicle pool reduced” connects the KEs from several AOPs, such as AOP IDs 398 and 238, and pAOPs 1, 2, 4, 5, and 6 with AOs “Irregularities, ovarian cycle” or “Impaired fertility”. The KE “Oocyte meiosis disrupted” connects two MIEs, “ALDH1A (RALDH) activation” and “CYP26B1 expression” with the reduction of ovarian follicle pool and impaired fertility, whereas the KE “Reduced E2 in circulation” connects the MIE “Aromatase inhibition” and the KE “Decreased ovarian E2” with the AO “Impaired fertility” through the KE “Irregularities, ovarian cycle”, and through the KE “Reduced ovulation”.

Another important score is the node eccentricity score, which sorts the KEs into upstream and downstream elements. In the directed networks, the farther downstream a KE is, it will show the greater eccentricity score (and the lower the inverse) (Spinu et al. 2019). In the HFRT-AOP network, the MIE “Inhibition of aromatase” showed the highest eccentricity score (score 6), followed by other MIEs in the network, such as “Antagonism, Androgen receptor”, “ALDH1A (RALDH) inhibition”, “Deposition of energy” (score 5). Some KEs including “Decreased ovarian E2” and “Decreased Kisspeptin release from neurons in AVPV” also show a higher eccentricity score (score 5) (Supplementary Material 1, Table S5). This score suggests that these nodes are farther downstream in the network. This is in line with their AO description of MIE. In the AOP network of human hepatotoxicity, nodes such as “Activation, SREBF1”, “Decreased, PPAR-beta activation” or “Binding of inhibitor, mitochondrial complex” that are defined as MIEs also showed the highest eccentricity scores (Arnesdotter et al. 2021). The convergent KEs and divergent KEs in the HFRT-AOP network are shown in Table 2.

**Table 2:** Convergent KEs and divergent KEs in the HFRT-AOP network

Convergent KEs		Divergent KEs	
KE type	KE name	KE type	KE name
AO	Impaired fertility	KE	Reduced E2 in circulation
AO	Irregularities, ovarian cycle	KE	Altered transcription of genes by AR
KE	Decreased all-trans retinoic acid (atRA) concentration	KE	Increased NR2F1 activation
KE	Accelerated follicle formation		
KE	Impaired oocyte maturation		

### Collection of the ovarian genes affected by DEHP exposure

We retrieved a total of 538 studies investigating the effect of DEHP in different organs and systems from the CTD (http://ctdbase.org). These studies included in vitro cell culture exposure experiments and in vivo animal studies. Most of the in vitro and in vivo studies describe the effect of DEHP exposure on male gonads/reproductive systems. However, we have focused our attention on the effect of DEHP on female ovaries; hence, the ovaries were selected as the target organ. After classification and screening, we have selected nine studies that describe the effect of DEHP on female ovaries. The selected studies from CTD are shown in Supplementary Material 2, Table S6.

We retrieved 71 DEHP target genes from the nine abovementioned studies. The most associated genes (2 or more times citied in association with DEHP) are aromatase (*CYP19A1*), BCL2 associated X, apoptosis regulator (*BAX*), BCL2 apoptosis regulator (*BCL2*), cyclin D2 (*CCND2*), cyclin dependent kinase 4 (*CDK4*), and c-kit proto-oncogene (*KIT*). The full list of genes affected by DEHP exposure in the ovaries can be found in Supplementary Material 2, Table S7.

### Identification of molecular initiating events and biological processes associated with DEHP-induced ovarian toxicity

Since modulation of the receptor activity could represent an MIE, we retrieved nuclear receptors that are affected by DEHP from the CompTox Chemicals Dashboard as of February 7, 2022 (https://CompTox.epa.gov/dashboard). The search revealed that ER A and B, nuclear receptor subfamily 1 group I member 2 (NR1I2), PPARG, and thyroid hormone receptor (THR) were modified by DEHP in various assays.

**Fig. 3:**
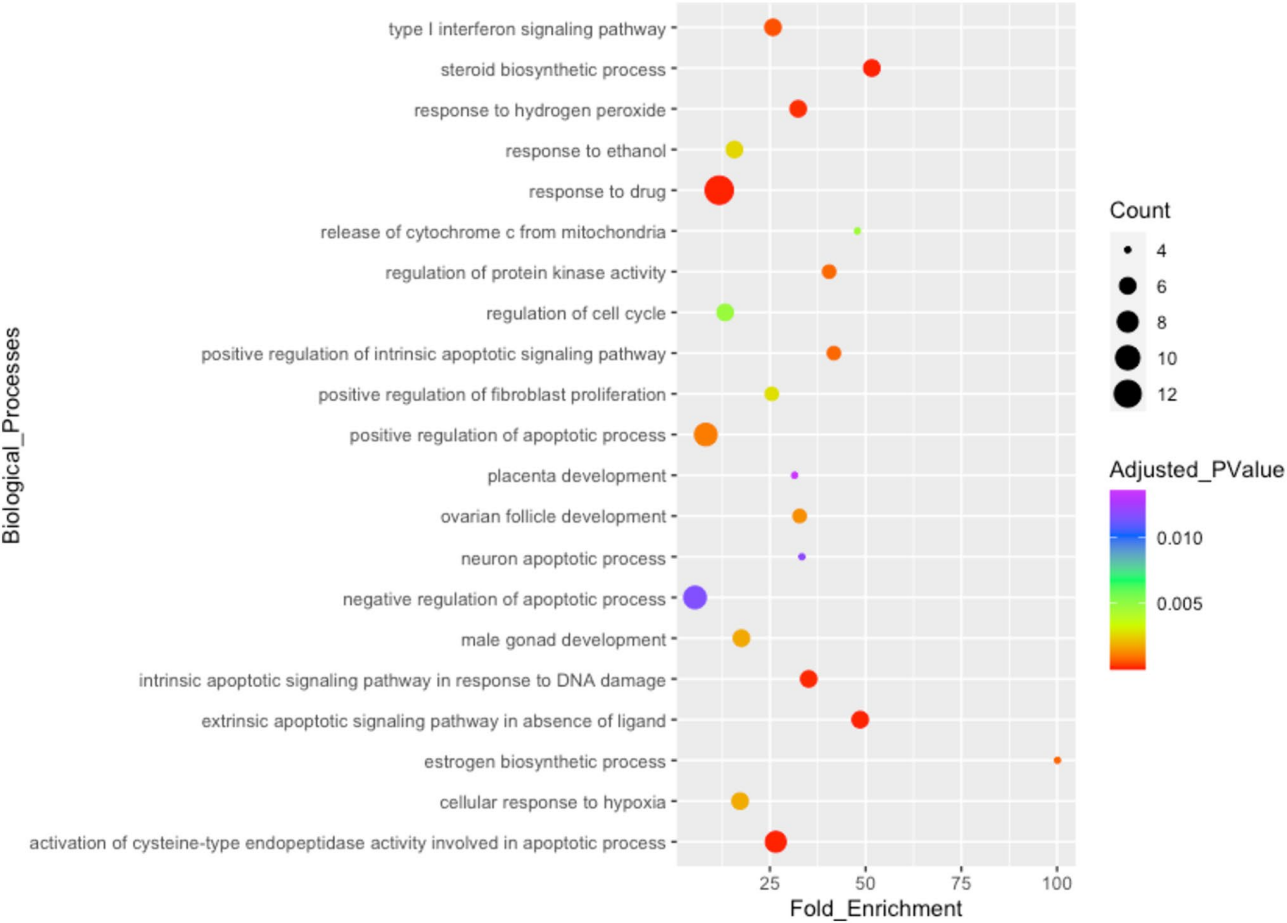
Biological processes associated with DEHP-affected genes in the ovaries

To extract the BPs, DEHP-affected genes in the ovaries were imported into the DAVID tool (https://david.ncifcrf.gov) and the enriched GO BPs were retrieved. The results of the GO enrichment analysis in DAVID demonstrate that BPs such as activation of the cysteine-type endopeptidase activity involved in the apoptotic process, steroid biosynthetic process, intrinsic apoptotic signaling pathway in response to DNA damage, positive regulation of intrinsic apoptotic signaling pathway, and estrogen biosynthetic process could be altered in DEHP-exposed ovaries (Fig. 3).

Automatic and manual screening of the literature was carried out to capture as much as possible of the existing information linking DEHP with MIEs and BPs in the ovary. First, we ran the AOP-helpFinder tool to search for the connection between DEHP and MIEs using DEHP synonyms and nuclear receptors identified from the CompTox Chemicals Dashboard. This search identified 182 publications co-mentioning DEHP with nuclear receptors. Further manual curation of the extracted publications identified PPARG and ER as being co-mentioned with DEHP in the ovary. The second literature search was conducted using AOP-helpFinder associations between the stressor DEHP and the BPs identified by DAVID. The analysis identified 259 publications, among them terms related to apoptosis (167 publications) and cell cycle (26) being the most co-mentioned with DEHP. Additional manual curation of the PubMed database retrieved publications describing the effect of DEHP in the ovary of rodents.

### Mapping DEHP to the HFRT-AOP network

**Fig. 4:**
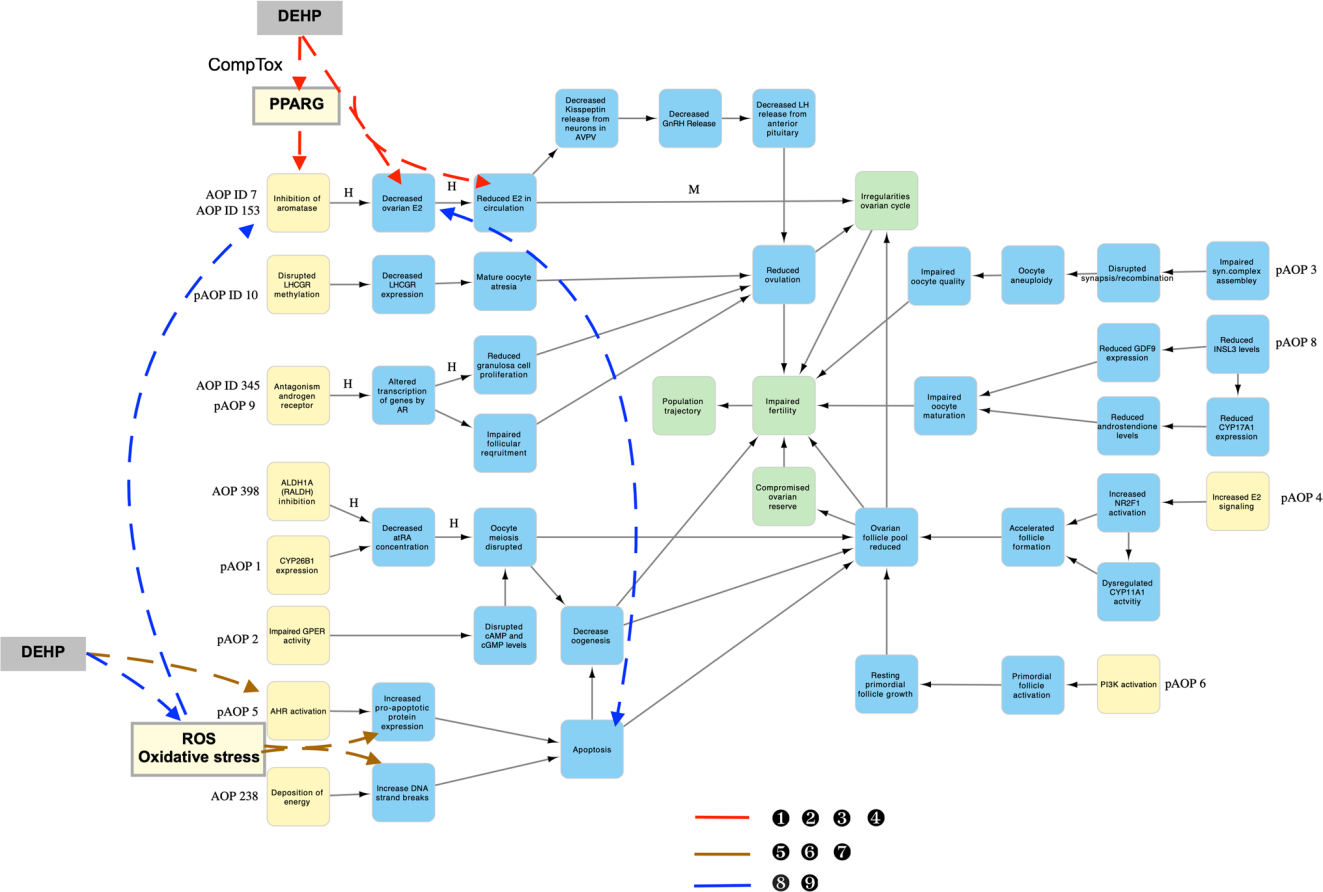
Representation of the HFRT-AOP network involving DEHP. Numbers near colored lines correspond to the scientific literature (please see Supplementary Material 2, Table S3)

Next, we combined all this information to map DEHP to the HFRT-AOP network (Fig. 4). Three different paths for DEHP-induced human female reproductive toxicity were mapped. The first pathway is related to E2 production, which is a part of the subnetwork “Reduced ovulation-dependent fertility impairment”. The MIE “Inhibition of aromatase” and the two KEs “Decreased ovarian E2” and “Reduced E2 in circulation” were shown to be affected by DEHP in experimental studies (Lai et al. 2017; Liu et al. 2021a; Tripathi et al. 2019a). Further support for the connection between DEHP exposure and E2 biosynthesis was provided by the KEGG analysis of DEHP-affected genes in which the estrogen biosynthetic process was identified as the key BP affected by DEHP exposure. Since DEHP is mapped to a divergent KE “Reduced E2 in circulation”, it can further branch its effect into two different AOs: (i) it can either lead to the AO “Impaired fertility” through a direct action on the ovaries, thus causing irregularities of the ovarian cycle, or (ii) it can act on the hypothalamus-hypophysis axis by decreasing the GnRH and LH release, thereby reducing the number of ovulated oocytes.

Another important consideration of DEHP action through E2 biosynthesis is its MIE. “Inhibition of aromatase” has been annotated as the MIE 408 in AOP ID 7 (AOP-Wiki); however, PPARG has also been postulated as a regulator of aromatase expression/activity in AOP ID 7 (AOP-Wiki) and in pAOP7 (Johansson et al. 2020), thereby placing this nuclear receptor as the potential MIE for the subnetwork “Reduced ovulation-dependent impaired fertility”. The data from a fluorescence assay of mRNA induction obtained from the CompTox Chemicals Dashboard reveal that DEHP can modulate PPARG with an AC50 of 38 µM. This AC50 is above the lower bound of cytotoxicity of DEHP reported in the CompTox database (3.215 µM); however, this cytotoxic limit has been obtained from the assays on certain cell types and cannot represent a general cytotoxic response to DEHP. It has been shown that DEHP cytotoxicity limit was 50 µM in human granulosa cell line KGN (Ernst et al. 2014) or equal and above 100 µM in rat ovarian granulosa cells (Tripathi et al. 2019a). Therefore, the AC50 of 38 µM for DEHP-induced activation of PPARG might have a biological significance for granulosa cells.

Taking that PPARG could be the MIE, this first path of DEHP-mediated effect on human female reproductive system starts with activation of PPARG, which reduces the aromatase expression/activity leading to the reduction in E2 production in the ovary and serum levels, which can further cause irregularities of the ovarian cycle either directly or indirectly through the hypothalamus-pituitary axis leading to reduced ovulation. Both the indirect and the direct path converge to the terminal AO “Impaired fertility”. Since this path encompasses the KE “Reduced ovulation” and the AO “Irregularities in ovarian cycle”, it is more relevant for the DEHP-mediated effect on mature females. In this derived AOP, the KEs occur in a biologically plausible order. It has been shown that PPARG can be a mediator of DEHP-induced effect on steroidogenesis (Ernst et al. 2014) and it is directly connected to the inhibition of aromatase expression and E2 production in human granulosa cells (Kwintkiewicz et al. 2010). Since granulosa cells are the major source of E2, reduction of this steroid in the ovary would decrease its plasma concentration. The disruption of PPARG has also been associated with later KEs and AOs such as ovarian dysfunction and female subfertility (Cui et al. 2002). The weight of evidence on the effect of DEHP through this path reveals weak to moderate dose–response and temporal concordance between KEs. The same DEHP concentration of 400 µM (Tripathi et al. 2019a) or 50 µM (Ernst et al. 2014) decreases aromatase expression and lowers E2 production in granulosa cells. Moreover, 20 µg DEHP/kg bw/day decreases E2 in the ovary and serum in mice (Liu et al. 2021a). However, some studies showed that human relevant concentration of DEHP (10 nM) effects the aromatase expression without changing the E2 production (Jin et al. 2019). A lack of DEHP-mediated effect on aromatase expression (Lai et al. 2017) and E2 production (Guerra et al. 2016), and increase in E2 biosynthesis (Laskey and Berman 1993) have also been demonstrated. Some of these inconsistencies could possibly be attributed to different measurements during different stages of the estrous cycle, exposure concentrations, routes of delivery, etc. Moreover, DEHP is a highly hydrophobic compound and the effective concentration in in vitro assays may be confounded by the laboratory contamination. In addition, it has been shown that DEHP can impact hypothalamus, thus providing some experimental evidence for its action through the indirect path. DEHP affects kisspeptin mRNA at 5 and 500 mg/kg bw/day causing irregularities in ovarian cycle; however, these two doses exert an opposite effect on the examined parameters (Yu et al. 2020). DEHP has a stimulatory effect on the GnRH level in hypothalamus at a wide range of doses, from 0.2 mg/kg bw/day (Shao et al. 2019) to 3000 mg/kg bw/day (Liu et al. 2016, 2014). An increase in the GnRH level was also associated with a decrease in serum E2 level following exposure to 1000 and 3000 mg DEHP/kg bw/day (Liu et al. 2014). However, these DEHP effects on hormone levels were rarely connected to any KEs related to ovarian dysfunction. Low E2 level was associated with the inhibition of ovulation in DEHP-exposed animals (Davis et al. 1994; Lovekamp-Swan and Davis 2003); however, whether DEHP-induced suppression of ovulation occurred through its effect on hypothalamus or through a direct effect on the ovary remains to be elucidated. Therefore, more studies are needed to establish a solid quantitative and temporally coherent linkage of DEHP exposure, PPARG (MIE), and E2 level (KE) to the subsequent terminal KE and AOs.

The second pathway of DEHP-induced human female reproductive toxicity is related to apoptosis since the KE “Increase, DNA strand breaks” and the divergent KE “Apoptosis” were shown to be affected by DEHP in experimental studies (Liu et al. 2017, 2019; Sun et al. 2018; Tripathi et al. 2019b). Moreover, the KEGG pathway analysis of DEHP-affected ovarian genes demonstrated a connection between this ED and several BPs involved in apoptosis, such as activation of the cysteine-type endopeptidase activity involved in apoptotic process, extrinsic apoptotic signaling pathway in the absence of ligand, intrinsic apoptotic signaling pathway in response to DNA damage, and others. Since KE “Apoptosis” belongs to the subnetwork “Folliculogenesis-dependent fertility impairment” and encompasses the KEs “Decrease oogenesis” and “Ovarian follicle pool reduced”, this pathway of DEHP action could be more relevant for the developing ovaries. The specific MIE involved in DEHP-induced disruption of folliculogenesis has not been determined. Two MIEs, namely “AHR activation” and “Deposition of energy”, are connected to apoptosis in the HFRT-AOP network. While “Deposition of energy” is referred to ionization in the AOP-Wiki and most likely does not represent an MIE, “AHR activation” could be an initial event leading to DEHP-induced apoptosis. It has been shown that DEHP (Ernst et al. 2014) and its major metabolite MEHP (Lovekamp-Swan et al. 2003) can increase mRNA expression of the aryl hydrocarbon receptor (AHR) in granulosa cells. However, a search of the CompTox database did not reveal the interaction of DEHP with any of the in vitro AHR assays. Possible indirect evidence for the connection between DEHP and AHR could represent the detection of reactive oxygen species (ROS) and oxidative stress in DEHP-exposed cultured newborn mouse ovaries (Liu et al. 2019), cumulus cells of large animals (Ambruosi et al. 2011), murine ovarian antral follicles (Wang et al. 2012), and rat granulosa cells (Tripathi et al. 2019a).

In the HFRT-AOP network, activation of the AHR is only connected with increases in pro-apoptotic protein expression. Being experimentally associated with DNA strand breaks in DEHP-exposed ovaries (Lu et al. 2019), oxidative stress can connect AHR with increase in DNA strand breaks. Increases in pro-apoptotic protein expression and increase in DNA strand breaks will lead to apoptosis, which will further cause a decrease in ovarian follicle pool and oogenesis and finally impaired fertility. This could represent the second possible AOP of DEHP in the female reproductive system. The overall assessment of this AOP reveals a rather moderate dose–response and temporal concordance between the KEs. There is a good dose–response and temporal concordance between DEHP, oxidative stress, DNA damage, apoptosis, and the oocyte or the follicle number. In murine oocytes, 10 µM DEHP (Liu et al. 2017) or 20 and 40 µg DEHP/kg bw/day (Liu et al. 2021a) causes DNA damage and apoptosis leading to reduced number of oocytes and follicles. Similarly, 40 μg DEHP/kg bw/day causes oxidative stress, DNA damage, and apoptosis, thus affecting fertilization capabilities of murine oocytes (Lu et al. 2019). Moreover, there is an association between DEHP exposure and later KE in this path, such as primary follicle assembly (Liu et al. 2021b; Mu et al. 2015). It can be argued that DEHP-mediated apoptotic effect could be the result of a general toxicity; however, studies have shown that apoptosis may either target only one group of cells in the follicle (Li et al. 2016) or it may be reversible (Sun et al. 2018), which speaks in favor of the specificity of DEHP-mediated apoptotic effect in the ovary. The association between DEHP exposure and the KE “Increases in the pro-apoptotic protein expression” is somewhat inconsistent and lacks dose-dependent and temporal evidence. DEHP at 20 µg/kg bw/day increases caspase expression and decreases cell proliferation (Liu et al. 2021a, b). Exposure to 10 µM DEHP causes apoptosis; however, an increase in the expression of the pro-apoptotic Bax gene is only observed with 100 µM DEHP (Zhang et al. 2014). Therefore, the weight of evidence to connect the AHR with oxidative stress and other KEs in this path needs to be further solidified.

The third pathway of DEHP in the HFRT-AOP network encompasses two KEs that belong to different subnetworks: “Reduced ovarian E2” and “Apoptosis”. This pathway of DEHP-induced human female reproductive toxicity is depicted in the study of Tripathi et al. (2019a) and involves ROS and oxidative stress formation, which decrease the expression of aromatase and reduce E2 production in the ovary leading to apoptosis in DEHP-exposed granulosa cells. Although this DEHP action could be a part of the above-explained pathways by which this ED affects the female reproductive system, we have decided to segregate it for the reasons described below. First, it provides a new experimentally determined connection between two KEs in different subnetworks: reduction of E2 in the ovary and apoptosis. A new connection established in the AOP network has the potential to reveal unknown relationships between distant KEs and may represent toxicity pathways specific to EDs. It may also lead to the prediction of unknown adverse effects upon specific ED exposure, as well as guide future development of new AOPs (Ravichandran et al. 2022). Such a new path, called an emergent path, can also lead to development of new stand-alone AOPs (Villeneuve et al. 2018). Therefore, the new connection established in the HFRT-AOP network may represent an emergent path in DEHP-induced female reproductive dysfunction. We have also found that this possible DEHP emergent path is supported by several lines of evidence showing a concomitant increase in oxidative stress and a decrease in E2 production in granulosa cells (Cui et al. 2022; Tabandeh et al. 2022; Tripathi et al. 2019a; Zaidi et al. 2021). Secondly, this pathway implicates oxidative stress as a potential MIE of DEHP-induced human female reproductive toxicity. In linear AOPs used to construct the HFRT-AOP network, ROS formation and oxidative stress were not annotated KE; however, the evidence demonstrate that ROS can affect the KE ”Inhibition of aromatase” (reference 9, Supplementary Material 2, Table S8) or promote the KE “Apoptosis” (references 5, 6, and 7, Supplementary Material 2, Table S8). Therefore, DEHP-induced ROS formation and oxidative stress should be investigated as the potential MIE for DEHP-induced reproductive dysfunction and the possible common MIE for the KEs related to E2 production (KE “Inhibition of aromatase”) and the KEs related to apoptosis (KEs “Increase, DNA strand breaks” and “Increased pro-apoptotic protein expression”). However, it is also possible that apoptosis induced by oxidative stress can reduce the number of active steroidogenic cells, thus resulting in a decrease in E2 production (Hannon et al. 2015). The exact sequence of events in this third pathway of DEHP action should be experimentally determined.

## Conclusion

A plethora of toxicological information is scattered in a cloud of various databases. Although obtained by using appropriate methodologies and endpoint measurements, these data may show inconsistencies in the selected exposure concentrations or routes of exposure. Sometimes, the mechanism of action is complex to the point that the obtained data is somewhat inconsistent and float around the threshold between the effect and the no effect. Integration of all evidence is required to understand all possible means by which chemicals can affect various levels of biological organization. Further quantification and evaluation of possible toxicity pathways, including dose- and time-resolved responses, could provide an emergent or plausible pathway(s) of chemicals action. Here, we proposed an AOP network for human female reproductive toxicity to provide a ground for better understanding of the mechanistic complexity underlying perturbation of the human female reproductive dysfunction. This network for human female reproductive toxicity was constructed using 15 individual AOPs. Although the majority of individual AOPs obtained from the AOP-Wiki (4 out of 5) have “under development” status and ten AOPs from the paper of Johansson et al. are not yet included in the AOP-Wiki, the status of these AOPs was not a limitation for their usage in the AOP network construction (Spinu et al. 2019). Since these 15 individual AOPs are causal constructions and putative, the proposed HFRT-AOP network is also causal and putative, which can affect the confidence of network models in predicting toxicity and chemicals’ safety. Despite these shortcomings, the HFRT-AOP could be a useful tool to predict the human female reproductive toxicity of chemicals in animal-fee settings. This is particularly important since numerous published data demonstrate that many chemicals may cause alterations in female reproductive behavior and contribute to subfecundity, infertility, pregnancy loss, growth retardation, intrauterine fetal demise, birth defect, and ovarian failure in laboratory animals and wildlife (Sharara et al. 1998). Furthermore, there is a large diversity of mechanisms involved in female reproductive toxicity, such as immune disruption, DNA adduct formation, altered cellular proliferation, and inappropriate cellular death (Sharara et al. 1998). To address some of these challenges, the development of the AOP network related to human female reproduction offers a potential opportunity to increase the efficiency of testing, generate predictive models, and link EDs to complex human female disorders across different biological levels of organization. The applicability of the HFRT-AOP network was exercised by mapping DEHP to the network, thus identifying divergent paths by which DEHP can cause female reproductive dysfunction. This approach has allowed for identification of potentially relevant MIEs, KEs, data gaps, and emergent paths where the experimental efforts should be focused to advance the mechanistic understanding of DEHP-induced female reproductive dysfunction.

## Supplementary Information

Below is the link to the electronic supplementary material.

Supplementary file 1 (XLSX 27 KB)

Supplementary file 2 (DOC 162 KB)
