# Supplementary material for: Mapping DEHP to the adverse outcome pathway network for human female reproductive toxicity
Source: Arch Toxicol. 2022 Jul 5;96(10):2799–813. doi: 10.1007/s00204-022-03333-y (PMC9352620; doi:10.1007/s00204-022-03333-y)
Supplement: Supplementary file 2 — (DOC 162 KB) [file 204_2022_3333_MOESM2_ESM.doc]

**SUPPLEMENTARY MATERIAL**

Kristina Pogrmic-Majkic*, Dragana Samardzija Nenadov, Biljana Tesic, Svetlana Fa,
Dunja Kokai, Bojana Stanic, Nebojsa Andric

University of Novi Sad, Faculty of Sciences, Department of Biology and Ecology, Serbia

**Supplementary Table S6.** Nine references related to DEHP-induced ovarian toxicity retrieved from the Comparative Toxicogenomics Database.

| **PubMed ID** | **Title** | **References** |
| --- | --- | --- |
| 29385306 | Di(2-ethylhexyl) phthalate (DEHP) influences follicular development in mice between the weaning period and maturity by interfering with ovarian development factors and microRNAs | (Liu et al., 2018) |
| 27405655 | Di (2-ethylhexyl) phthalate impairs steroidogenesis in ovarian follicular cells of prepuberal mice | (Lai et al., 2017) |
| 26678702 | Acute exposure to di(2-ethylhexyl) phthalate in adulthood causes adverse reproductive outcomes later in life and accelerates reproductive aging in female mice | (Hannon et al., 2016) |
| 25701202 | Di(2-ethylhexyl) phthalate inhibits antral follicle growth, induces atresia, and inhibits steroid hormone production in cultured mouse antral follicles | (Hannon et al., 2015) |
| 24804967 | Daily exposure to Di(2-ethylhexyl) phthalate alters estrous cyclicity and accelerates primordial follicle recruitment potentially via dysregulation of the phosphatidylinositol 3-kinase signaling pathway in adult mice | (Hannon et al., 2014) |
| 22155089 | Di (2-ethylhexyl) phthalate inhibits growth of mouse ovarian antral follicles through an oxidative stress pathway | (Wang et al., 2012) |
| 22279657 | Effects of di-(2-ethylhexyl) phthalate exposure on reproductive development and PPARs in prepubertal female rats | (Ma et al., 2011) |
| 19874833 | Di-(2-ethylhexyl) phthalate and mono-(2-ethylhexyl) phthalate inhibit growth and reduce estradiol levels of antral follicles *in vitro* | (Gupta et al., 2010) |
| 16763069 | Exposure of prepubertal female rats to inhaled di(2-ethylhexyl)phthalate affects the onset of puberty and postpubertal reproductive functions | (Ma et al., 2006) |

**Supplementary Table S7.** The list of 71 genes relevant to DEHP-induced ovarian toxicity extracted from nine references retrieved from the Comparative Toxicogenomics Database.

| **Gene Symbol** | **Gene Name** |
| --- | --- |
| *ALDH3A2* | Aldehyde dehydrogenase 3 family member A2 |
| *ATM* | ATM serine/threonine kinase |
| *B2M* | Beta-2-microglobulin |
| *BAD* | BCL2 associated agonist of cell death |
| *BAX* | BCL2 associated X, apoptosis regulator |
| *BCL2* | BCL2, apoptosis regulator |
| *BCL2L10* | BCL2 like 10 |
| *BOK* | BOK, BCL2 family apoptosis regulator |
| *C3* | Complement C3 |
| *CASP3* | Caspase 3 |
| *CASP8* | Caspase 8 |
| *CAT* | Catalase |
| *CCNA2* | Cyclin A2 |
| *CCNB1* | Cyclin B1 |
| *CCND2* | Cyclin D2 |
| *CCNE1* | Cyclin E1 |
| *CD74* | CD74 molecule |
| *CDK4* | Cyclin dependent kinase 4 |
| *CDKN1A* | Cyclin dependent kinase inhibitor 1A |
| *COL4A6* | Collagen type IV alpha 6 chain |
| *CYP11A1* | Cytochrome P450 family 11 subfamily A member 1 |
| *CYP17A1* | Cytochrome P450 family 17 subfamily A member 1 |
| *CYP19A1* | Cytochrome P450 family 19 subfamily A member 1 |
| *FCGR2B* | Fc fragment of igg receptor iib |
| *FGGY* | FGGY carbohydrate kinase domain containing |
| *GDF9* | Growth differentiation factor 9 |
| *HEPHL1* | Hephaestin like 1 |
| *HJURP* | Holliday junction recognition protein |
| *HK3* | Hexokinase 3 |
| *HSD17B1* | Hydroxysteroid 17-beta dehydrogenase 1 |
| *HSD17B2* | Hydroxysteroid 17-beta dehydrogenase 2 |
| *HSD3B1* | Hydroxy-delta-5-steroid dehydrogenase, 3 beta- and steroid delta-isomerase 1 |
| *IFIT1* | Interferon induced protein with tetratricopeptide repeats 1 |
| *IFIT2* | Interferon induced protein with tetratricopeptide repeats 2 |
| *INHBB* | Inhibin beta B subunit |
| *IRF7* | Interferon regulatory factor 7 |
| *ISG15* | ISG15 ubiquitin-like modifier |
| *ITGA2* | Integrin subunit alpha 2 |
| *JCHAIN* | Joining chain of multimeric iga and igm |
| *KIT* | KIT proto-oncogene receptor tyrosine kinase |
| *LAMC2* | Laminin subunit gamma 2 |
| *LDLR* | Low density lipoprotein receptor |
| *LHB* | Luteinizing hormone beta polypeptide |
| *LHCGR* | Luteinizing hormone/choriogonadotropin receptor |
| *MGLL* | Monoglyceride lipase |
| *MTOR* | Mechanistic target of rapamycin |
| *MX2* | MX dynamin like gtpase 2 |
| *OAS2* | 2'-5'-oligoadenylate synthetase 2 |
| *PDPK1* | 3-phosphoinositide dependent protein kinase 1 |
| *PPARG* | Peroxisome proliferator activated receptor gamma |
| *PTEN* | Phosphatase and tensin homolog |
| *RPS6* | Ribosomal protein S6 |
| *S100A9* | S100 calcium binding protein A9 |
| *SETD3* | SET domain containing 3 |
| *SNORA30* | Small nucleolar RNA, H/ACA box 30 |
| *SOD1* | Superoxide dismutase 1 |
| *STAC* | SH3 and cysteine rich domain |
| *STAR* | Steroidogenic acute regulatory protein |
| *SYNDIG1* | Synapse differentiation inducing 1 |
| *TAP1* | Transporter 1, ATP binding cassette subfamily B member |
| *TSC1* | Tuberous sclerosis 1 |
| *USP18* | Ubiquitin specific peptidase 18 |
| *RIPOR3* | RIPOR family member 3 |
| *KITL* | KIT ligand |
| *ADH1* | Alcohol dehydrogenase 1 |
| *AKR1B7* | Aldo-keto reductase family 1 |
| *GPX* | Glutathione peroxidase |
| *FCGR4* | Fc receptor, IgG, low affinity IV |
| *TRIM30D* | Tripartite motif-containing 30D |
| *IFI27L2A* | Interferon, alpha-inducible protein 27 like 2A |

**Supplementary Table S8.** The list of 9 manually selected references from the PubMed used to map DEHP to the HFRT-AOP network.

| **No** | **Reference** | **Type of experiments and end points** |
| --- | --- | --- |
| 1 | (Ernst et al., 2014) | *In vitro* human granulosa cells – E2 level |
| 2 | (Kwintkiewicz et al., 2010) | *In vitro* in human granulosa cells – E2 level |
| 3 | (Liu et al., 2021) | *In vivo* - mother and the suckling mice – E2 level in ovary and serum, DNA damage and apoptosis |
| 4 | (Lai et al., 2017) | *In vivo* prepubertal mice – E2 level in ovary |
| 5 | (Liu et al., 2017) | Cultured fetal mouse oocyte – DNA damage, apoptosis, reduced oocyte survival and follicle assembly |
| 6 | (Liu et al., 2019) | Cultured newborn ovaries – ROS, DNA damage, apoptosis, and primordial follicle assembly |
| 7 | (Sun et al., 2018) | Cultured fetal mouse ovaries – DNA double-strand breaks, ROS formation, and apoptosis |
| 8 | (Tripathi et al., 2019) | *In vitro* rat granulosa cells – E2 level, ROS formation, and apoptosis |
| 9 | (Hannon et al., 2015) | Cultured antral follicled - E2 level, atresia, apoptosis |
|  | | |

**Supplementary References**

Ernst, J., Jann, J.C., Biemann, R., Koch, H.M., Fischer, B., 2014. Effects of the environmental contaminants DEHP and TCDD on estradiol synthesis and aryl hydrocarbon receptor and peroxisome proliferator-activated receptor signalling in the human granulosa cell line KGN. Molecular human reproduction 20, 919-928.

Gupta, R.K., Singh, J.M., Leslie, T.C., Meachum, S., Flaws, J.A., Yao, H.H., 2010. Di-(2-ethylhexyl) phthalate and mono-(2-ethylhexyl) phthalate inhibit growth and reduce estradiol levels of antral follicles in vitro. Toxicol Appl Pharmacol 242, 224-230.

Hannon, P.R., Brannick, K.E., Wang, W., Gupta, R.K., Flaws, J.A., 2015. Di(2-ethylhexyl) phthalate inhibits antral follicle growth, induces atresia, and inhibits steroid hormone production in cultured mouse antral follicles. Toxicol Appl Pharmacol 284, 42-53.

Hannon, P.R., Niermann, S., Flaws, J.A., 2016. Acute Exposure to Di(2-Ethylhexyl) Phthalate in Adulthood Causes Adverse Reproductive Outcomes Later in Life and Accelerates Reproductive Aging in Female Mice. Toxicological sciences : an official journal of the Society of Toxicology 150, 97-108.

Hannon, P.R., Peretz, J., Flaws, J.A., 2014. Daily exposure to Di(2-ethylhexyl) phthalate alters estrous cyclicity and accelerates primordial follicle recruitment potentially via dysregulation of the phosphatidylinositol 3-kinase signaling pathway in adult mice. Biol Reprod 90, 136.

Kwintkiewicz, J., Nishi, Y., Yanase, T., Giudice, L.C., 2010. Peroxisome proliferator-activated receptor-gamma mediates bisphenol A inhibition of FSH-stimulated IGF-1, aromatase, and estradiol in human granulosa cells. Environmental health perspectives 118, 400-406.

Lai, F.N., Liu, J.C., Li, L., Ma, J.Y., Liu, X.L., Liu, Y.P., Zhang, X.F., Chen, H., De Felici, M., Dyce, P.W., Shen, W., 2017. Di (2-ethylhexyl) phthalate impairs steroidogenesis in ovarian follicular cells of prepuberal mice. Arch Toxicol 91, 1279-1292.

Liu, J., Wang, W., Zhu, J., Li, Y., Luo, L., Huang, Y., Zhang, W., 2018. Di(2-ethylhexyl) phthalate (DEHP) influences follicular development in mice between the weaning period and maturity by interfering with ovarian development factors and microRNAs. Environ Toxicol 33, 535-544.

Liu, J.C., Lai, F.N., Li, L., Sun, X.F., Cheng, S.F., Ge, W., Wang, Y.F., Li, L., Zhang, X.F., De Felici, M., Dyce, P.W., Shen, W., 2017. Di (2-ethylhexyl) phthalate exposure impairs meiotic progression and DNA damage repair in fetal mouse oocytes in vitro. Cell Death Dis 8, e2966.

Liu, J.C., Li, L., Yan, H.C., Zhang, T., Zhang, P., Sun, Z.Y., De Felici, M., Reiter, R.J., Shen, W., 2019. Identification of oxidative stress-related Xdh gene as a di(2-ethylhexyl)phthalate (DEHP) target and the use of melatonin to alleviate the DEHP-induced impairments in newborn mouse ovaries. J Pineal Res 67, e12577.

Liu, J.C., Xing, C.H., Xu, Y., Pan, Z.N., Zhang, H.L., Zhang, Y., Sun, S.C., 2021. DEHP exposure to lactating mice affects ovarian hormone production and antral follicle development of offspring. J Hazard Mater 416, 125862.

Ma, M., Kondo, T., Ban, S., Umemura, T., Kurahashi, N., Takeda, M., Kishi, R., 2006. Exposure of prepubertal female rats to inhaled di(2-ethylhexyl)phthalate affects the onset of puberty and postpubertal reproductive functions. Toxicological sciences : an official journal of the Society of Toxicology 93, 164-171.

Ma, M., Zhang, Y., Pei, X., Duan, Z., 2011. [Effects of di-(2-ethylhexyl) phthalate exposure on reproductive development and PPARs in prepubertal female rats]. Wei Sheng Yan Jiu 40, 688-692, 697.

Sun, Z.Y., Zhang, P., Wang, J.J., Liu, J.C., Li, L., Shen, W., Zhai, Q.Y., 2018. Melatonin alleviates meiotic defects in fetal mouse oocytes induced by Di (2-ethylhexyl) phthalate in vitro. Aging (Albany NY) 10, 4175-4187.

Tripathi, A., Pandey, V., Sahu, A.N., Singh, A., Dubey, P.K., 2019. Di-(2-ethylhexyl) phthalate (DEHP) inhibits steroidogenesis and induces mitochondria-ROS mediated apoptosis in rat ovarian granulosa cells. Toxicology research 8, 381-394.

Wang, W., Craig, Z.R., Basavarajappa, M.S., Gupta, R.K., Flaws, J.A., 2012. Di (2-ethylhexyl) phthalate inhibits growth of mouse ovarian antral follicles through an oxidative stress pathway. Toxicol Appl Pharmacol 258, 288-295.
